# Relevance of Eosinophilia and Hyper-IgE in Immigrant Children

**DOI:** 10.1097/MD.0000000000000043

**Published:** 2014-06-04

**Authors:** Moncef Belhassen-García, Javier Pardo-Lledías, Luis Pérez del Villar, Antonio Muro, Virginia Velasco-Tirado, Ana Blázquez de Castro, Belen Vicente, Mª Inmaculada García García, Juan Luis Muñoz Bellido, Miguel Cordero-Sánchez

**Affiliations:** Servicio de Medicina Interna, Sección de Enfermedades Infecciosas (MB-G); Servicio de Medicina Interna, Hospital General de Palencia “Río Carrión,” Palencia (JP-L); IBSAL, CIETUS (LPDV); Laboratorio de Inmunología Parasitaria y Molecular, Facultad de Farmacia (AM); Servicio de Medicina Interna (VV-T); Servicio de Microbiología, Grupo de Investigación Reconocido MICRAPE (ABDC); Laboratorio de Inmunología Parasitaria y Molecular, Facultad de Farmacia (BV); Servicio de Microbiología (MIG); Servicio de Microbiología, Grupo de Investigación Reconocido MICRAPE (JLMB); Servicio de Medicina Interna, Seccion de Enfermedades Infecciosas (MC-S), Complejo Asistencial Universitario de Salamanca (CAUSA), Instituto de Investigación Biomédica de Salamanca (IBSAL), Centro de Investigación de Enfermedades Tropicales de la Universidad de Salamanca (CIETUS), Universidad de Salamanca, Salamanca, Spain.

## Abstract

Immigrants from undeveloped countries are a growing problem in Europe. Spain has become a frequent destination for immigrants (20% of whom are children) because of its geographic location and its historic and cultural links with Africa and Latin America. Eosinophilia is frequent in adult immigrants, travelers and expatriates coming from tropical areas. However, there are few studies that focus on the incidence and causes of tropical eosinophilia and hyper-IgE in immigrant children.

We evaluated, prospectively, the prevalence and causes of eosinophilia and hyper-immunoglobulin E (IgE) in 362 immigrant children coming from Sub-Saharan Africa, Northern Africa and Latin America to Salamanca, Spain, between January 2007 and December 2011.

Absolute eosinophilia and hyper-IgE were present in 22.9% and 56.8% of the analyzed children, respectively. The most frequent causes of absolute eosinophilia were filariasis (52.6%), strongyloidiasis (46.8%) and schistosomiasis (28.9%). Filariasis (41.9%), strongyloidiasis (29.6%) and schistosomiasis (22.2%) were the most frequent causes of increased levels of IgE. The area under the ROC curve showed similar values between eosinophil count and IgE levels in the diagnosis of helminthiasis (69% [95% confidence interval (CI) 63%–74%] vs 67% [95% CI 60%–72%], *P* = 0.24)*.*

Eosinophilia and hyper-IgE have a high value as biomarkers of helminthiasis in children coming from tropical and subtropical areas.

## INTRODUCTION

The migratory flow from developing to developed countries is continuously increasing. Therefore, imported infectious diseases and the health status of immigrant population has become a relevant subject in developed countries.^1,2^ Helminthiasis, such as filariasis and schistosomiasis, are an important cause of the disease burden, affecting 20% and 6% of immigrants coming from endemic areas, respectively.^2,3^ However, specific symptoms associated with these parasitic infections are frequently absent.

Several studies have documented the relationship between eosinophilia, hyper-immunoglobulin E (IgE) and helminthiasis in immigrant adults and travelers coming from tropical areas.^4–6^ In fact, both parameters are considered as biomarkers for parasitic infections. However, data about imported diseases focusing on immigrant children are scarce and no data on the prevalence and causes of eosinophilia and hyper-IgE in immigrant children coming from tropical and subtropical areas are available.^7^

The main objective of the present study is to know the prevalence and causes of eosinophilia and hyper-IgE in immigrant children coming from Sub-Saharan Africa, Northern Africa and Latin America areas and to describe the usefulness of eosinophilia and hyper-IgE as biomarkers for parasitic infection.

## PATIENTS AND METHODS

The study was carried out in the Tropical Medicine Unit (TMU), Complejo Asistencial Universitario de Salamanca (CAUSA), Salamanca, Spain. We evaluated, prospectively, the prevalence and causes of eosinophilia and hyper-IgE in immigrant children coming from tropical or subtropical areas between January 2007 and December 2011. The study was reviewed and approved by the Ethical Committee of the CAUSA and the written consent was obtained from legal guardians. The criteria for inclusion were as follows: 1) age less than 18 years and 2) immigrants from Sub-Saharan Africa, North Africa and Latin America**.**

A defined set of demographic, clinical and laboratory data was collected for each immigrant children. *Children recently arrived*: defined as less than 6 months of stay. *Children with long-term stay*: defined as more than 6 months of stay. *Eosinophilia*: defined as an increase in peripheral blood eosinophilic leukocytes to more than 0.450 × 10^9^ eosinophils/L of blood.^6^ *Hyper-IgE*: defined as an increase in peripheral blood IgE more than 100 U/mL.^8^ It is classified as mild (≥100–399 U/mL), moderate (≥399–999 U/mL) and severe (≥1000 U/mL).

Indirect parasitological tests eincluded commercial serologic tests for *Echinococcus granulosus* and *Taenia solium*. Home-made serological enzyme-linked immunosorbent assay manufactured with adult whole worm antigens of *Dirofilaria immitis*^9^ and *Schistosoma bovis*,^5^ excretion/secretion antigens of *Fasciola hepatica*^10^ and somatic larvae antigens of *Strongyloides venezuelensis* were used for diagnosis of filariasis, schistosomiasis, fasciolosis and strongyloidiasis, respectively. Direct parasitological tests included the examination of 3 stool samples, and in selected cases, the following were also used: 1) optic microscopy of a terminal urine specimen and/or 2) Knott test for microfilaremia and/or 3) skin snips.

One-way analysis of variance test was used for comparing eosinophil count and IgE levels among patients with single and multiple infections, the 3 pediatric age groups (0–7 y, 7–14 y and 14–18 y) and 3 origin areas (Sub-Saharan Africa, North Africa and Latin America). Pearson’s correlation was computed to identify the relationship between IgE levels and eosinophil count. Meanwhile, the χ^2^ and the Fisher exact tests were used to test associations between categorical variables, specifically to evaluate the association between demographic variables and final diagnoses. The level of statistical significance accepted was *P* < 0.05 and the results were expressed as mean plus standard deviation (SD).

Receiver-operating-characteristic (ROC) curves were obtained by plotting the sensitivity versus 1–specificity for the full range of eosinophils and IgE levels cutoff points for both cases and controls to estimate the cutoff value that had the highest overall validity. “Sensitivity” was calculated as the number of individuals positive for serologic tests and eosinophils and IgE levels at different breakpoints divided by the total number of cases. “Specificity” was considered as the number of individuals negative for serologic tests and negative for eosinophils and IgE levels at different breakpoints divided by the total number of controls. The area under the ROC curve was calculated by determining the probability of correctly identifying (accuracy) a randomly selected participant as either a case (serologic test positive) or a noncase (serologic test negative). The 45-degree line in each ROC curve graph subsumes an area equal to 0.5 (50%). Using the ROC curves, an optimal breakpoint was determined for the eosinophils counts and IgE levels that maximized the sensitivity and specificity in classifying an individual at serological positive to helminthic infection. Based on this cutoff point, the positive predictive value (PPV) and negative predictive value (NPV) of eosinophils counts and IgE levels to detect helminthic infections were determined.

Data were entered into an Access database. After internal consistency checks, all statistical analyses and data visualization were performed in R 3.0.0 (R Foundation for Statistical Computing; http://cran.r-project.org/). ROC curves were analyzed and visualized using ROC R package.^11^

## RESULTS

### Patient Characteristics

The study included 362 children coming from tropical and subtropical areas. Most children came from Sub-Saharan Africa [242/362, (66.8%)], followed by Northern Africa [67/362, (18.5%)] and Latin America [53/362, (14.6%)]. The home countries of children included in the study are shown in Table 1. The mean age of children was 12.3 ± 4.0 years; 171 (47.2%) were girls and 47.9% of these patients had arrived recently. North African immigrants were most frequently asymptomatic (*P* < 0.05). Combined symptoms (digestives and cutaneous) were most frequently associated to Sub-Saharan patients and other symptoms to Latin American patients (*P* < 0.05). The baseline demographic and main analytical and clinical features of the enroled participants, categorized by origin areas, are shown in Table 2.

**TABLE 1:**
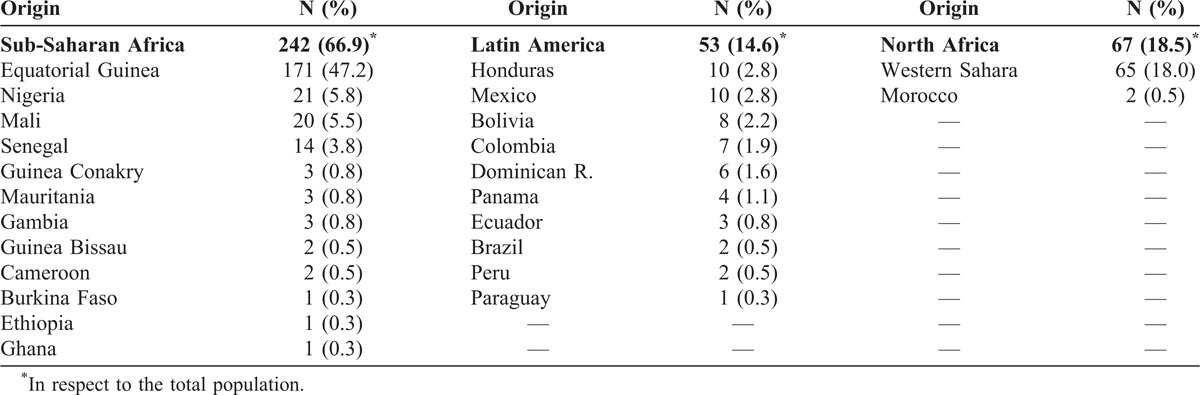
Country of Immigrant Children Included in the Study

**TABLE 2:**
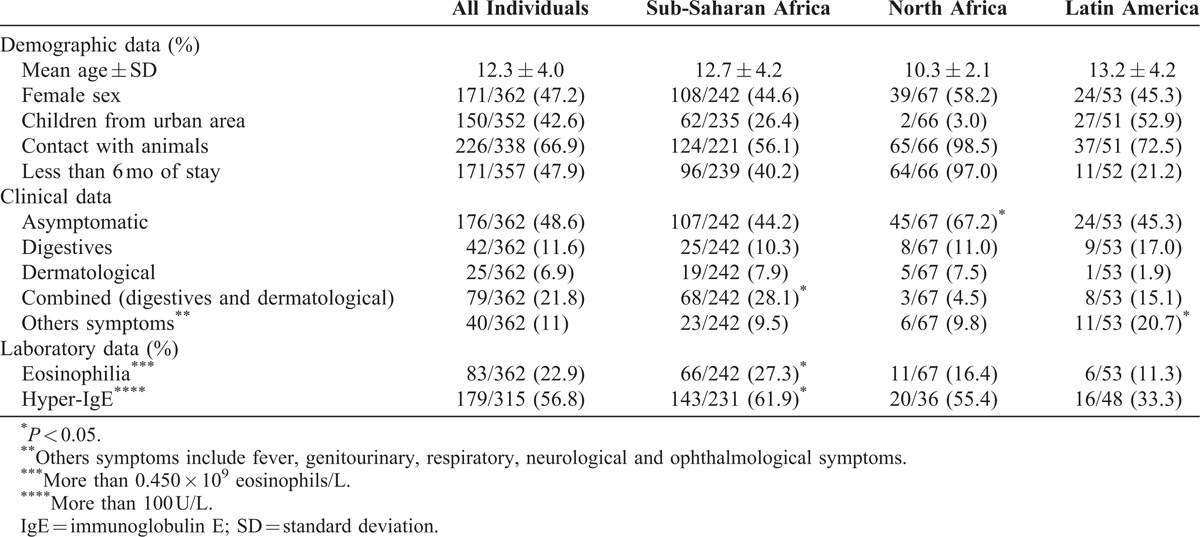
Demographic, Clinical and Laboratory Data of Immigrant Children

### Eosinophilia and Hyper-IgE in Immigrant Children

Eighty three out of 362 (22.9%) immigrant children analyzed had absolute eosinophilia: 50 (13.8%) children had mild eosinophilia (≥0.45–0.99 × 10^9^ eosinophils/L), 23 (6.1%) had moderate eosinophilia (≥0.99–2.99 × 10^9^ eosinophils/L) and 10 (2.8%) had severe eosinophilia (≥3.00 × 10^9^ eosinophils/L); 36 children with absolute eosinophilia (43.4%) were asymptomatic and 47 children (56.6%) had symptoms. The most frequent symptoms were combined digestive and cutaneous symptoms [21, (25.3%)], followed by digestives symptoms alone [10, (12.0%)] and skin lesions alone [5, (6.0%)]. Only 4 patients (2.2%) had asthma or atopic/allergic symptoms. Three children with eosinophilia were taking drugs unrelated to eosinophilia and all of them had helminthiasis.

Regarding hyper-IgE severity, 91/315 (43.2%) children had mild levels of hyper-IgE (≥100–399 U/mL), 45/315 (14.3%) had moderate levels of hyper-IgE (≥399–999 U/mL) and 43/315 (13.7%) had severe levels of hyper-IgE (≥1000 U/mL); 81 children (45.3%) with high levels of IgE were asymptomatic. Among the 98 (54.7%) symptomatic patients, the most frequent clinical features were combined digestive and cutaneous symptoms [43, (24%)], digestive symptoms [20, (11.2%)] and skin lesions [14, (7.8%)].

Among the 315 patients with increased IgE levels, eosinophilia was also detected in 69 (21.9%). Statistical tests showed a significant association between eosinophilia and hyper-IgE (odds ratio = 8.7, CI 3.9%–19.5%, *P* < 0.001).

**FIGURE 1:**
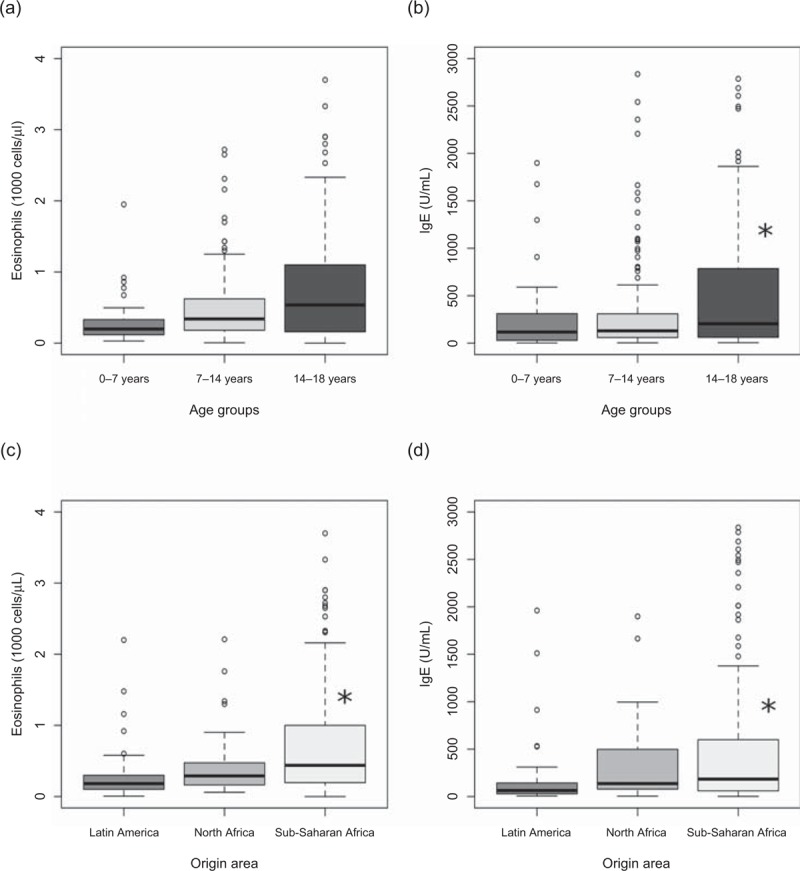
Relationship between eosinophils counts and age of individuals (A) and origin (C). Relationship between IgE levels and age of individuals (B) and origin (D). IgE = immunoglobulin E.

We further analyzed the relationship between demographic variables, eosinophils count and IgE levels. Eosinophils counts tend to increase with the age of individuals (Figure 1A), although no significance was reached among age groups. IgE levels showed the same trend. IgE levels of patients aged 14–18 years were significantly higher, as compared with patients aged 0–7 years ([703.0 U/L] vs [264.0 U/L], *P* < 0.05) (Figure 1B). Both eosinophils counts ([0.533 × 10^9^ eosinophils/L] vs [0.122 × 10^9^ eosinophils/L], *P* < 0.05) (Figure 1C) and IgE levels ([588.2 U/L] vs [184.0 U/L], *P* < 0.05) (Figure 1D) were significantly higher in children coming from Sub-Saharan Africa as compared with children coming from Latin America. There were no significant differences of IgE levels or eosinophils counts between children who arrived recently and who stayed for long term, or between children coming from rural and urban areas.

### Eosinophilia and Hyper-IgE Caused by Helminthiasis

In order to analyze the causes of eosinophilia and hyper-IgE, parasitic infections were actively searched by both serological and direct methods**.** Significant increases of eosinophils counts ([0.709 × 10^9^ eosinophils/L] vs [0.226 × 10^9^ eosinophils/L], *P* < 0.05) and IgE levels ([743.9 U/L] vs [299.2 U/L], *P* < 0.05) were observed in patients with a final diagnosis of helminthiasis compared with patients who had not this final diagnosis.

**FIGURE 2:**
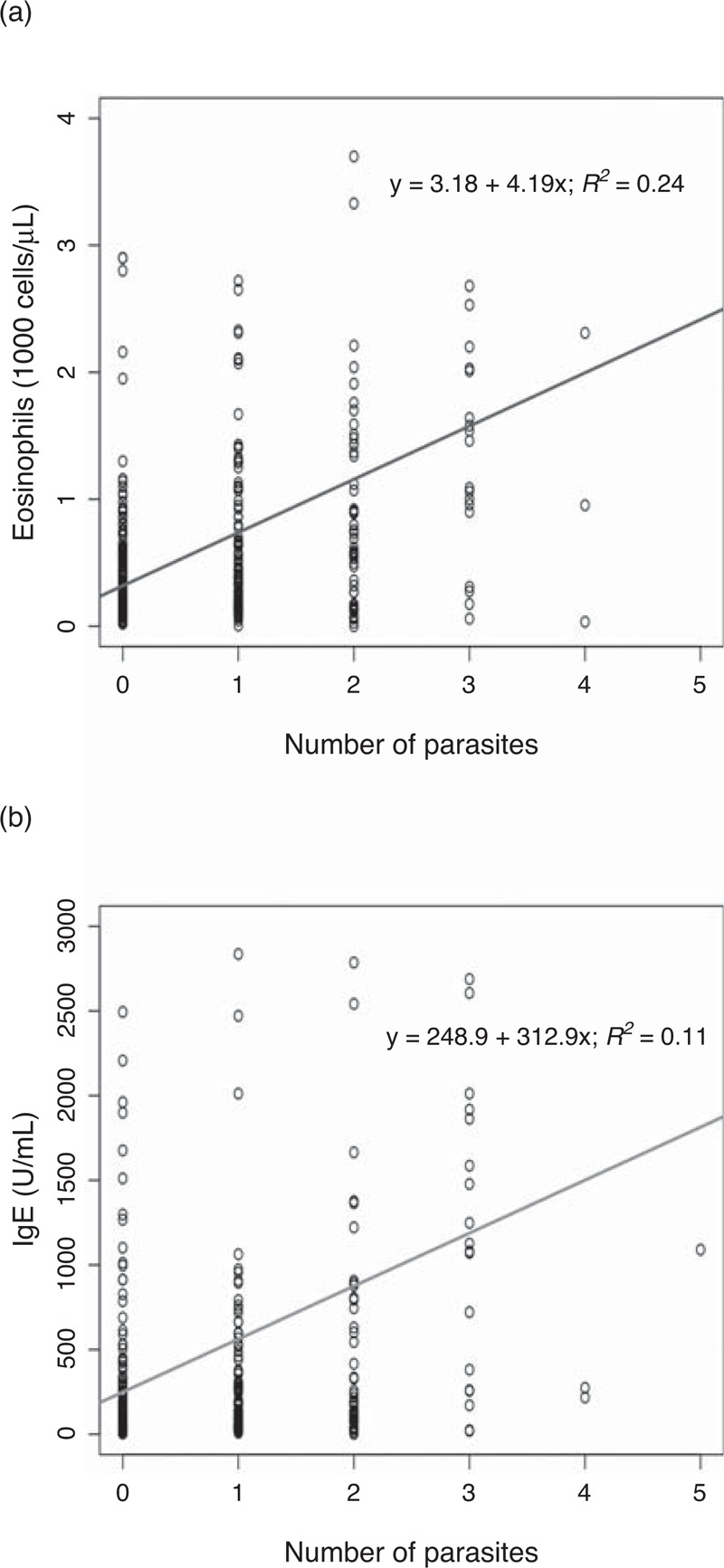
(A) Relationship between number of parasites and eosinophils counts. (B) Relationship between number of parasites and IgE levels. IgE = immunoglobulin E.

A final diagnosis of absolute eosinophilia was found in 64 (77.1%) cases; 27 of them (32.5%) were infected by 1 parasite species, and 37 (44.6%) were infected with 2 or more parasite species. In the same way, among the 111 patients with abnormal IgE levels, 56 (31.3%) patients had 1 parasite species and 55 (30.7%) patients had 2 or more parasite species. We described the relationship between IgE, eosinophils and number of parasites through linear regression adjustment (Figure 2). In both cases, we observed that both the eosinophils counts and the IgE levels increased significantly with the number of parasite species found (*P* < 0.05). Regarding the parasite species identified, the most frequent causes of absolute eosinophilia were filariasis (52.6%), strongyloidiasis (46.8%) and schistosomiasis (28.9%). Filariasis (41.9%), strongyloidiasis (29.6%) and schistosomiasis (22.2%) were the most frequent causes of increased levels of IgE (Table 3).

**TABLE 3:**
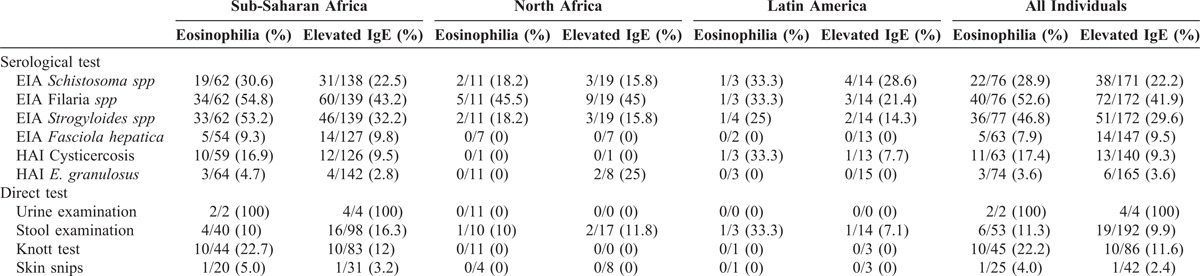
Direct and Serological Parasitological Test in Patients with Eosinophilia and High IgE Levels

### Eosinophilia and Hyper-IgE as Biomarkers of Helminthic Infections in Immigrant Children

**FIGURE 3:**
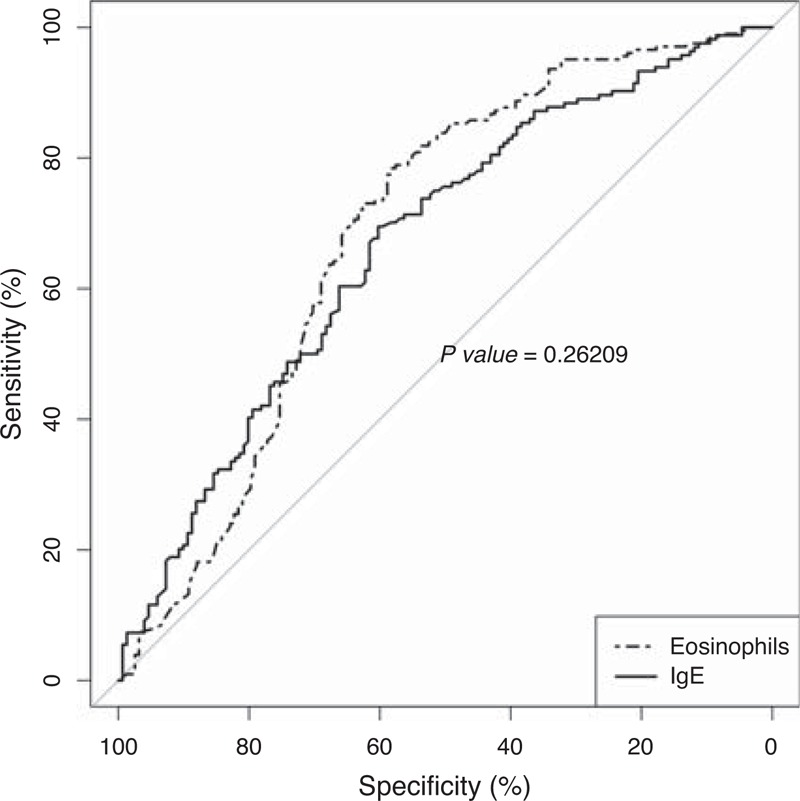
Area under the ROC curve (AUCs) of the eosinophilia and IgE levels as biomakers of helminthic infections.

We used area under the ROC curve (AUCs) as indication of the “accuracy” of the eosinophilia and IgE levels as biomarkers of helminthic infections (Figure 3). For the optimal cutoff of 0.266 × 10^9^ eosinophils/L and 167 U/L of IgE levels, the area under the ROC curve showed similar values between eosinophils count and IgE levels in the diagnosis of helminthiasis (69% [95% CI 63%–74%] vs 67% [95% CI 60%–72%], *P* = 0.24), respectively. In fact, there was a weak correlation between eosinophils count and IgE levels (*r* = 0.272, *P* < 0.05). For the established cutoff greater than 0.45 × 10^9^ eosinophils/L, the area under the ROC curve, which is an indication of the “accuracy” of the test or proportion of all tests that have reported the correct result, was 65% (95% CI 63%–74%) for helminthiasis. Concerning IgE levels, for a cutoff of 100 U/L, the proportion of all tests that have reported the correct result for helminths infections was 65% (95% CI 60%–72%). Table 4 shows the sensitivity (S), specificity (Sp), PPV, NPV and ABC ROC (95%) for eosinophilia and high levels of IgE as biomarkers for the diagnosis of helminthic infections.

**TABLE 4:**
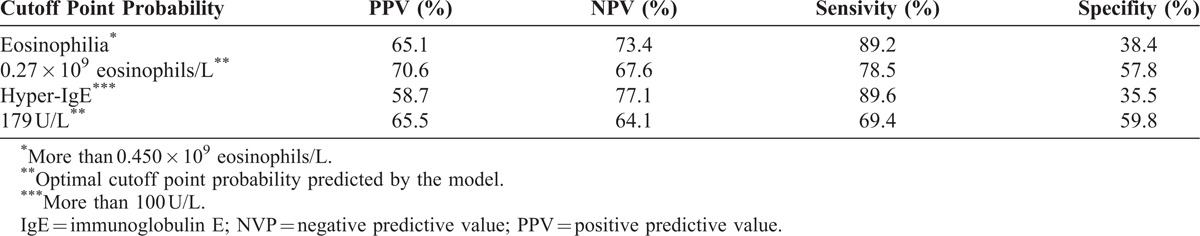
Diagnostic Value of the Helminthiasis for Different Cutoff Points for Eosinophils Count and IgE Levels

## DISCUSSION

In the last decade, several reports focusing on health status of immigrant population coming from tropical and subtropical areas have been published. Health problems in this population can be diverse^12^ but infectious diseases, including latent tuberculosis, hepatitis B, HIV and, mainly, parasitic diseases are the most frequent diseases detected in immigrant population.^2^ Information available about the health status of immigrant children is limited.^13–15^ Only a few reports evaluating health requirements of immigrant children have been published.^16^ To our knowledge, there is no data about the prevalence of parasitic disease and its association with IgE or eosinophilia biomarkers in widely available, international journals.

Imported eosinophilia is a frequent finding in adult immigrants arrived from tropical and subtropical countries. Its prevalence ranges from 10% to 50%, depending on the cutoff established and the origin area, and is more frequent in immigrants from Sub-Saharan Africa or Southeast Asia.^17,18^ In a previous publication, we studied a group of Sub-Saharan African adults and found eosinophilia in 27% of them.^6^

We focused this study on immigrant children referred to the TMU of CAUSA for illness or for routine screening. Most children came from Africa, mainly, Sub-Saharan Africa, and a lower percentage from Latin America. The whole prevalence of eosinophilia was 22.9%, higher than rates found in other studies. Seybolt et al^13^ in a retrospective study focusing on a refugees group comprising a wide range of age (2 mo to 81 y, mean age 17.3 y) found a prevalence of eosinophilia of 12%. In other retrospective study focusing on children mainly coming from Southeast Asia, Dawson-Hahn et al^16^ detected a eosinophilia rate of 19%. In our study, eosinophilia prevalence was higher in African (mainly, Sub-Saharan) children. This result agrees with the published in imported eosinophilia either in adult^4^ or in children.^16^

Eosinophilia may emerge in adults in a wide range of disorders, including immunological disorders, drugs hypersensitivity, hematological and neoplastic disorders and infectious diseases, especially those caused by parasites. Parasitic infections, especially helminthiasis, are the most frequent cause of eosinophilia worldwide^19^ and is the main cause of eosinophilia in travelers, immigrants and expatriates coming from tropical and subtropical areas.^5,7,17,18,20–22^

There is a very short number of studies published about the causes of eosinophilia in children.^23^ In Western countries, hypersensitivity to food and inhaled antigen are a frequent cause of autochthonous eosinophilia. Immunological disorders, adverse drug reactions, hematological and neoplastic disorders and idiopathic diseases, such as eosinophilic colitis or vasculitis, are also the causes of eosinophilia as mentioned in the literature. Concerning parasitic diseases, the most frequent parasite identified in our area as a cause of eosinophilia is the visceral larva migrans. In fact, in a recent study developed in Croatia, 31% of children with asymptomatic eosinophilia were seropositive for *Toxocara canis.*^24^ These results highlight that toxocariasis should be included in the differential diagnosis of eosinophilia, especially in children.

As happens for adult population, the causes of eosinophilia depend on the origin of children. This way, Seybolt et al^13^ found a high seroprevalence for *Filaria* (51%), *Strongyloides* (39%) and *Schistosoma* (22%) in a group coming from Africa, while Dawson-Hahn et al^16^ detected a lower seropositivity for *Strongyloides* (22%) and *Schistosoma* (8%) in children coming mainly from Southeast Asia.

We prospectively investigated the administration of drugs to children associated to eosinophilia, and we did not detect any patient receiving any drugs frequently associated to eosinophilia. Only 3 patients had clinical data of asthma, rhinitis or hypersensitivity. This finding agrees with other studies in Africans children, which also find a prevalence of asthma around 3%.^25^ Although we did not use any specific immunological test, no children showed clinical data suggesting the presence of immunological diseases associated to eosinophilia. On the contrary, using a diagnostic algorithm previously validated in adults with eosinophilia,^6,26^ we found that more than 75% of patients presented with parasitic infections, mainly, helminthiasis. Other authors have also mentioned this inverse relationship between a high rate of parasitic/helminthic infection and low frequency of asthma or atopic dermatitis.^27–29^ Although the cause is unknown, a strong polyclonal IgE immune response stimulated by helminthes, resulting in mast or basophiles cell receptors to specific allergens blockade is a feasible possibility. In this respect, van den Biggelaar et al^27^ proved that helminth-induced interleukin-10 lowers the hypersensitivity to allergens.

The most frequent causes of eosinophilia in our study were *Schistosoma spp*, filaria species and geohelminthic infections. This fact is most likely associated to the origin of patients, mainly coming from Sub-Saharan Africa.^7,13,21^ A remarkable fact is that intestinal parasites were detected in the stool study of only 11.3% of children with eosinophilia. We shall have in account that half of the patients were long-term stay children, and previous studies have shown that the possibility of intestinal parasites direct detection decreases with time.^4^

A limitation of our work was the lack of immunological tests for diagnosis of “visceral larva migrans" caused by *Toxocara canis*. Toxocariosis is frequent in children in tropical and subtropical areas, although its seroprevalence shows a wide range of variation (4%–56%).^30^ Moreover, toxocariasis causes eosinophilia in above 70% of cases.^29^ Probably, some undiagnosed cases of eosinophilia might be associated to this parasite.^31^ A second limitation is the possibility of cross-reactions in serological test.^32,33^

We found a linear correlation between the eosinophilia level and coparasitation. Patients parasitized by a higher number of different species had a higher eosinophilia level. We also detected a higher eosinophilia in patients with filarial infection, as compared with other parasitic infections. This finding has been previously referred by others.^6,13^

The value of eosinophilia as a biomarker for helminthiasis is not well resolved. In our article, we compared this biomarker with IgE levels, another biomarker frequently used in the clinical of tropical diseases. Using the cutoff established by the manufacturer, we detected that hyper-IgE prevalence was twice the eosinophilia prevalence. As happened with eosinophilia, we detected a correlation between high IgE levels and filarial infection or coparasitation. This fact reflects the important role of both biomarkers in the development of Th2 immune response against helminthiasis.

We used ROC curves for comparing eosinophilia and IgE as biomarkers for imported helminthiasis in children with a high probability pretest. The area under the ROC curve was next to 0.7, higher in eosinophilia than in IgE, although differences were not significant. When we used the cutoff of eosinophilia and hyper-IgE previously established (>0.450 × 10^9^ eosinophils/L and >100 UI respectively), the PPV was higher for eosinophilia than for IgE increase, and the contrary happened to NPV. There are no studies published using ROC curve for eosinophilia and IgE evaluation in helminthiasis. Data from articles published about this topic show a PPV of 14%–75% and NPV of 84%–98%.^6,17,21,26^ In a recent study in Brazil, patients with eosinophilia and IgE increase had relative risks for helminthiasis of 11 and 8, respectively.^34^ These results show the relative high value of eosinophilia and IgE as biomarkers for helminthiasis in patients from tropical and subtropical areas. It is important to highlight that data about the value of these biomarkers should be considered only in patients with a high probability pretest.

The present study has some limitations. First, the patients included in our study, probably, are not a representative sample of the whole group of minor immigrant population in Spain in terms of origin area, since most immigrant minors in Spain come from Northern Africa and Latin America. Most Sub-Saharan African patients included in our study came from a collaboration agreement between TMU and different associations hosting immigrant children, which usually work with Sub-Saharan minors. Second, the high infection rate in Africa, especially with respect to tropical diseases, makes more likely that immigrants from this continent have an infectious disease and, therefore, should be referred to the TMU. Thus, the frequencies of diagnoses may be biased by the imbalance in areas of origin of the patients. Third, the study may also be biased because they are children treated in a specific consultation of tropical diseases. For these reasons, the rates of diseases that we have found in this study should be analyzed and interpreted with caution before extrapolating them to the general minor immigrant population.

In summary, eosinophilia and hyper-IgE are frequent in immigrant children coming from Sub-Saharan Africa, North Africa and Latin America areas, even if they are asymptomatic, and both data have a similar high value as biomarkers for helminthiasis. The most frequent causes were filariasis, strongyloidiasis and schistosomiasis.
